# Sustained complete remission of human epidermal growth factor receptor 2-positive metastatic breast cancer in the liver during long-term trastuzumab (Herceptin) maintenance therapy in a woman: a case report

**DOI:** 10.1186/1752-1947-4-401

**Published:** 2010-12-10

**Authors:** John Syrios, Anna Dokou, Nicolas Tsavaris

**Affiliations:** 1 Medical Oncology Unit, Department of Pathophysiology, Laikon General University Hospital, Athens University School of Medicine, Athens 11527, Greece

## Abstract

**Introduction:**

This case report and short review discusses how long trastuzumab should be continued in metastatic breast cancer, the safety issues in case of pregnancy and the risk of relapse with trastuzumab cessation.

**Case presentation:**

We present the case of a 34-year-old Caucasian woman with human epidermal growth factor receptor 2-positive metastatic breast cancer in the liver who achieved prolonged complete remission within six months of receiving trastuzumab (Herceptin) in combination with vinorelbine and gemcitabine. The patient remains in complete remission seven years later and continues to receive trastuzumab as maintenance therapy.

**Conclusion:**

Trastuzumab-based therapies have greatly improved the survival rates of patients with human epidermal growth factor receptor 2- positive metastatic breast cancer. Despite such improvements, the safety of trastuzumab administration during pregnancy is yet to be defined.

## Introduction

The development of trastuzumab (Herceptin), a humanized human epidermal growth factor receptor 2 (HER2) monoclonal antibody, has changed the natural history of HER2-positive breast cancer, providing superior survival benefits in combination or as monotherapy compared with nontrastuzumab-based therapy [1,2]. However, HER2-positive metastatic breast cancer (MBC) is an aggressive disease, and despite these advances, the majority of patients treated with trastuzumab-based regimens progress within one year, with only very few patients experiencing prolonged remission [3].

The case report presented here describes a woman who underwent a mastectomy for invasive ductal carcinoma and subsequently received trastuzumab in combination with chemotherapy as treatment for a single metastatic lesion in the liver. She experienced a complete response, with disappearance of the hepatic lesion, and has been receiving maintenance trastuzumab for seven years. While taking trastuzumab, the patient expressed her intention of starting a family, which raised a number of questions, such as how long maintenance trastuzumab should be administered and whether, in this case, treatment should cease.

## Case presentation

In February 2001, an otherwise healthy 34-year-old Caucasian woman, with no history of hormone therapy, smoking, drinking, or a family history of breast cancer, presented with a lump in the center of her right breast. The axillary and neck lymph nodes were not palpably enlarged.

After breast biopsy and computed tomography (CT) of the chest and abdomen, the patient underwent a radical mastectomy. Pathologic examination of the resected specimens diagnosed HER2-positive (immunohistochemistry 3+), hormone receptor-negative, grade III, invasive ductal carcinoma of the right breast with two positive axillary lymph nodes. The size of the primary tumor was 4.3 × 5.5 × 3 cm. She was treated with sequential adjuvant chemotherapy, four cycles of epirubicin (75 mg/m^2^) followed by four cycles of docetaxel (70 mg/m^2^). On completion of chemotherapy in August 2001, radiation therapy was administered to the right breast.

**Figure 1:**
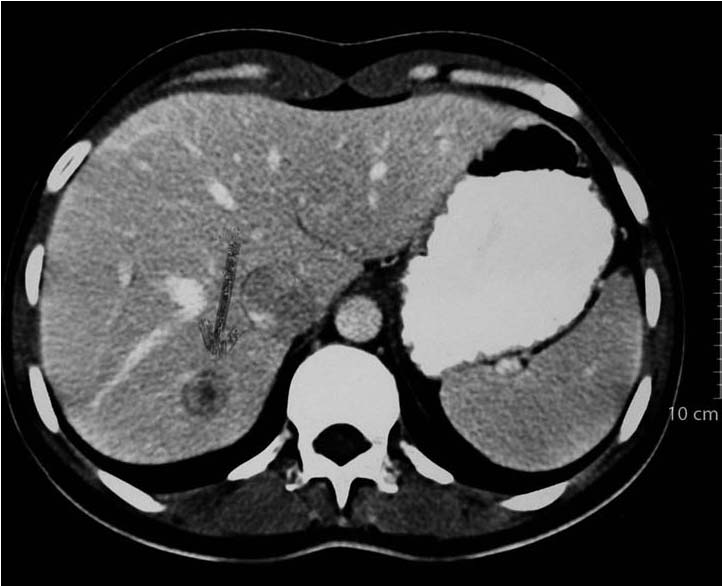
**Computed tomography scan of metastatic lesion in the liver taken in 2002**.

In January 2002, after CT, the patient presented with a single metastatic lesion (diameter, 1.4 cm) in the right lobe at segment 7 of the liver (Figure 1); no biopsy was carried out because our patient was unwilling to undergo such a procedure. Trastuzumab (4 mg/kg loading dose and 2 mg/kg weekly thereafter) in combination with 5-fluorouracil-leucovorin-methotrexate (600 mg/m^2^, 100 mg/m^2^, and 60 mg/m^2^, respectively) was started as first-line metastatic therapy in February 2002 for four months. The chemotherapy regimen was then changed to 40 mg/m^2 ^vinorelbine plus 600 mg/m^2 ^gemcitabine on days one and eight in a 21-day cycle and continued until April 2003.

**Figure 2:**
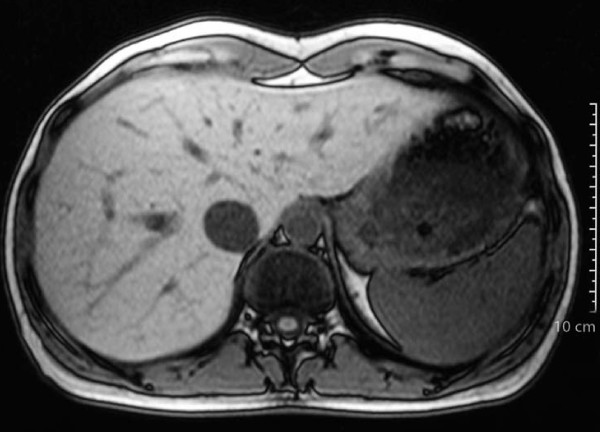
**Magnetic resonance imaging scan taken in 2003**. The hepatic lesion has disappeared.

**Figure 3:**
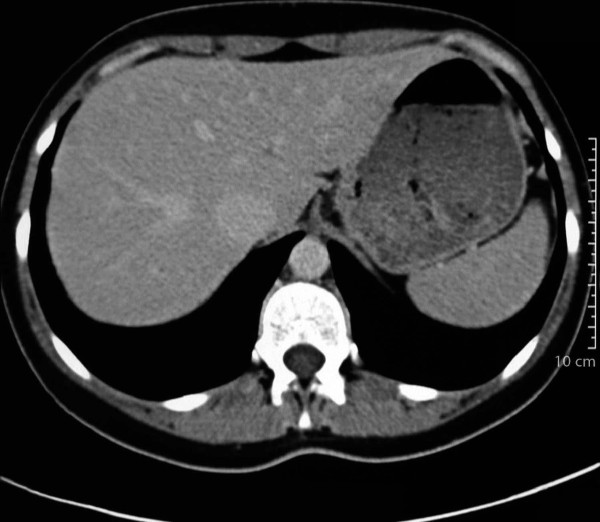
**Liver computed tomography scan after trastuzumab maintenance therapy**. The lesion has not reappeared.

Reevaluation of the lesion by ultrasonography and CT followed regularly thereafter and showed a complete response with disappearance of the hepatic lesion within six months. The complete response was affirmed by magnetic resonance imaging in December 2003 (Figure 2). On completion of the chemotherapy regimen, the patient continued to receive maintenance trastuzumab monotherapy (6 mg/kg every three weeks), and she remains in complete remission on maintenance trastuzumab (Figure 3).

Throughout this period, the patient was in good health and led an active life. In 2006, she decided that she would like to start a family, given that she had regular menses after completion of the adjuvant therapy. However, after being informed about the possible risks of trastuzumab treatment during pregnancy and the chance of relapse after trastuzumab withdrawal, she decided to continue treatment and not try for children. She has now had surgical breast reconstruction.

## Discussion

Clinical management of MBC remains a significant therapeutic challenge as oncologists balance improvements in overall survival with patients' quality of life [4]. Despite more than 30 years of research, MBC remains essentially incurable, with a median survival time of approximately two years [4]. The prognosis is poorer in patients with HER2-positive MBC [5]. Trastuzumab-based therapies have greatly improved the survival rates of these patients, with the largest benefits seen when treatment is continued at least until disease progression [1]. Despite such improvements, the safety of trastuzumab during pregnancy is yet to be defined.

Our patient wished to start a family; however, trastuzumab is classified as a category B drug and has been linked to a reduction in amniotic volume (anhydramnios) and fetal growth [6]. Thus, we would not encourage patients to continue trastuzumab while pregnant. However, if trastuzumab is withdrawn, there is the possibility that disease may relapse. Preclinical data suggest that previously suppressed tumor growth resumes rapidly if trastuzumab is withdrawn [7]. Effective treatment of HER2-positive disease therefore seems to require prolonged attenuation of HER2 activity, and it is difficult to define a time point beyond which trastuzumab might not offer additional benefit. Furthermore, evidence in the literature supports the idea that continuing anticancer treatments as maintenance therapy in patients in remission or with stable disease may prolong the disease-free interval [8].

There is an increasing number of case reports describing patients who experienced long-term remission from HER2-positive MBC while receiving trastuzumab maintenance therapy [9,10]. The duration of remission in these cases ranges from four months to eight years, and in all cases, maintenance therapy was based on trastuzumab. One of these cases also illustrates the risk of withdrawing trastuzumab treatment when the patient had experienced three years of full remission in the liver but relapsed in the central nervous system within two months of withdrawal of trastuzumab maintenance therapy [9].

Support for the importance of maintaining HER2 suppression is also provided by studies evaluating the use of trastuzumab beyond progression. Accumulating evidence shows the benefits of trastuzumab beyond progression, and it has been observed that progressive disease is not indicative of resistance to trastuzumab [11-14]. Clinically relevant objective responses to multiple lines of trastuzumab have consistently been observed in a multitude of prospective and retrospective analyses [12-14]. Additionally, in studies that have compared overall survival rates, significant improvements have been reported in patients continuing trastuzumab-based therapy beyond progression compared with those who stopped therapy [11,12]. However, because none of these studies were randomized, there could be a selection bias associated with the data; thus, randomized controlled studies are required to confirm the benefit of trastuzumab in this disease setting. Recently, the results from a phase III randomized study (GBG-26) comparing trastuzumab and capecitabine (Xeloda) with capecitabine alone in patients who progressed during trastuzumab therapy were reported [15]. Objective response rates were nearly doubled (48% vs. 27%), and time to progression extended (8.2 vs. 5.6 months) by the addition of trastuzumab to capecitabine, compared with capecitabine alone, without any unexpected toxicity [15].

An important concern of many clinicians regarding long-term use of trastuzumab is cardiac tolerability owing to the unexpected high incidence of cardiac events reported by the early pivotal trials, particularly when associated with anthracyclines. It is difficult to compare trials with different end points and eligibility criteria; however, the understanding of trastuzumab-related cardiac events has since improved, and the majority of these events are manageable and reversible. Extending trastuzumab treatment does not appear to be associated with an increased risk of cardiac dysfunction. In studies of trastuzumab treatment beyond progression, cardiac events appear to be relatively uncommon and mostly asymptomatic [13-15].

## Conclusion

Beyond our clinical experience, we predict that a number of patients (not reported) experience prolonged remission while receiving trastuzumab maintenance therapy. We propose that the molecular profile of a tumor and its biological environment, as governed by the specific traits of a patient, will influence whether a patient achieves long-lasting remission on maintenance trastuzumab therapy. We also speculate that the specific localization of breast cancer metastases may be a factor given that many of the cases reported to date are mainly associated with liver metastases [9,10]. Why this might be contributory needs additional investigation.

## Abbreviations

CT: computed tomography; HER2: human epidermal growth factor receptor 2; MBC: metastatic breast cancer.

## Competing interests

The authors declare that they have no competing interests.

## Consent

Written informed consent was obtained from the patient for publication of this case report and accompanying images. A copy of the written consent is available for review from the Editor-in-Chief of this journal.

## Authors' contributions

JS collected and analyzed the data of the study and wrote the manuscript. AD collected the data of the patient, and NT conceived and designed the study and supervised the manuscript writing. All authors read and approved the final manuscript.
